# Risks and protection: a qualitative study on the factors for internet addiction among elderly residents in Southwest China communities

**DOI:** 10.1186/s12889-024-17980-6

**Published:** 2024-02-20

**Authors:** Dan Wang, Xinyi Liu, Kun Chen, Chunyan Gu, Hongyan Zhao, Yong Zhang, Yu Luo

**Affiliations:** 1 School of Nursing, Army Medical University, Third Military Medical University, No. 30 Gaotanyan Street, Shapingba District, Chongqing, 400038 China; 2 Shuangbei Community Health Service Center, No.14 Shuangbei Street, Shapingba District, Chongqing, 400030 China; 3 Xiaolongkan Community Health Service Center, No.4 Xiaolongkan Street, Shapingba District, Chongqing, 400030 China

**Keywords:** Older adults, Internet addiction, Risk factors, Protective factors, Qualitative research

## Abstract

**Background:**

In the global trend of actively promoting the participation of older adults in the digital age, the relevant negative issues featuring potential Internet Addiction (IA) among them has risen to be a new challenge facing the global public health. However, there is a severe lack of related research. This study aimed to gain a comprehensive understanding of the phenomenon and process of IA among the elderly. The purpose of this paper is to introduce factors that may influence IA in the demographic.

**Methods:**

This study employed qualitative descriptive research methods to investigate older adults’ perceptions and experiences of IA. Semi-structured in-depth personal interviews were conducted between March and June 2023 with 36 senior citizens from urban communities in Chongqing, Southwest China. Data were analyzed via inductive content analysis methods.

**Results:**

Through data analysis, 2 main categories concerning IA in older adults were identified: risk factors and protective factors. The risk factor categories include 5 individual factors (e.g., Internet as the major avenue for pursuing personal hobbies and interests, etc.), 3 family factors (e.g., household WIFI increasing the risk of prolonged Internet use indoors, etc.), 2 peer factors (e.g., peer recommendation and guidance as catalysts for intensified Internet engagement, etc.), 2 socio-environmental factors (e.g., the widespread daily Internet use spurs offline intolerance, etc.), and 3 Internet platform factors (e.g., the plenitude of online content triggers endless viewing/browsing behaviors, etc.). The category of protective factors encompasses 2 individual factors (e.g., a higher level of perceived risk regarding internet health hazards, etc.) and 2 family factors (e.g., more family commitment, etc.).

**Conclusions:**

Older adults’ Internet addictive behaviors are shaped by multiple and complex internal and external factors. A higher level of online health risk perception is a key protective factor to effectively avoid the occurrence and deterioration of IA among the aged, a distinct finding from this study. It is believed that the “individual-family-peer-community” synergy strategy is expected to become an essential direction for IA intervention for older adults, in order to promote healthy Internet use among older adults.

## Background

The convergence of digitization and aging has become an important trend in the current international community. Internet usage among older adults has increased dramatically in recent years. For example, the internet usage rate among elderly individuals in the United States has risen from 12% in 2000 to 77.5% in 2018 [1]. Similarly, in Switzerland, it has also climbed from 10% in 2000 to 73% in 2017 [1]. In China, one of Internet giants with the largest number of Internet users in Asia and the world, the Internet use by seniors aged 60 and above has reached nearly 160 million by the end of 2022 [2, 3]. Against this backdrop, the potential negative problems related to IA among the elderly are also gradually posing a looming threat to global public health [4].

Consensus on the terminology and definitions related to IA has yet to be reached internationally. Concepts such as “Problematic Internet Use,” “Pathological Internet Use,” “Internet Use Disorders,” and “Excessive Internet Use” differ in their emphasis [5]. For instance, some scholars emphasize the negative consequences or harm resulting from internet usage when defining IA [6, 7], whereas other researchers highlight key behavioral characteristics of IA such as withdrawal symptoms, tolerance, and impulse control disorders [8, 9]. This study preliminarily delineates the concept of IA by integrating these various focuses: the negative impact on physical, psychological, and social aspects due to internet usage; or an inability to disengage from the internet or discomfort when away from it (withdrawal symptoms); or a need for increasingly more time spent online to satisfy the urge (tolerance); or an inability to resist the impulse to use the internet, despite the severe personal consequences that may ensue (impulse control disorders). Furthermore, in tandem with a more measured and controlled engagement with the internet, a broader segment of the populace might encounter adverse or problematic effects [10], suggesting an increasing normalization and prevalence of IA across individuals [11].

Recent systematic reviews and meta-analyses indicate that the overall prevalence rate of IA amongst the general population, predominately composed of adolescents, university students, healthcare professionals, and medical students, is approximately 14% [12]. Although there is a current lack of reported prevalence rates specifically pertaining to the elderly demographic, the emerging issues of IA among the cohort have been reported by several countries in recent years [1, 13–16], and shown to be associated with various negative health outcomes, such as lower levels of well-being, feelings of loneliness, depression, anxiety, psychological distress, social isolation, reduced social support, impaired sleep, and compromised daily functioning [1, 17–21]. Meanwhile, in Chinese society, there has been an endless influx of news reports regarding the detrimental effects of elderly addicted to the internet, including compromised physical health, disrupted daily routines, distorted perception of online information, and falling victim to Internet scams. Additionally, the South Korean government has recognized older adults as a high-risk and potentially risky group associated with smartphone addiction in 2019 [18]. That being said, the necessity and urgency of conducting research on IA among older adults require no further explanation.

However, existing research on IA has predominantly focused on younger populations such as children and adolescents, leaving a significant gap in our understanding of IA among older adults. Limited research on IA among older adults has been scattered across various factors influencing IA [13, 18, 20, 22]. Additionally, these studies have primarily used assessment tools designed for younger generations, which may not accurately reflect the characteristics and levels of IA among older adults. Unlike younger generations who grew up in the digital age, older adults experienced the advent of the digital era in adulthood, resulting in different patterns of digital use. For example, the high usage rates of smartphones among older adults cannot be directly compared to those of younger populations [23]. This highlights the need for specifical research focused on IA among older adults.

Previous studies have primarily focused on exploring the association between IA and personality traits, as well as the psychological factors involved [24]. These personality traits include low self-esteem, impulsivity, and neuroticism, while negative emotions such as depression, anxiety, loneliness, and escapism have also been examined. Additionally, comorbidities with mental disorders, such as attention-deficit/hyperactivity disorder (ADHD), suicidal or self-harm tendencies, risky behaviors, and eating disorders, have been investigated in relation to IA [24]. In addition, other risk factors include socio-environmental dimensions like problematic peer relationships, poor parental relationships, and family functioning [25]. In comparison, research on protective factors for IA is very limited. A recent scoping review identified three elements to prevent or mitigate IA, namely social support, level of engagement, and Internet self-efficacy [26]. However, currently, there is still a lack of clarity regarding the factors associated with IA among the elderly population.

Qualitative research plays a crucial role in describing addictive phenomena, identifying the process of addiction, and elucidating the perspectives of individuals with addiction [27]. Therefore, the objective of this research was to explore the phenomenon and process of IA among the elderly. This study generated content related to the influencing factors, behavioral characteristics, attitudes, and suggestions for improvement strategy associated with the phenomenon. However, the primary objective of this paper is to elucidate the factors influencing IA among older adults. This will provide a better understanding of IA behaviors among the elderly and serve as a foundation and reference for government authorities and professionals in developing scientifically sound assessment and intervention strategies for IA in the elderly population.

## Methods

### Design

This research adopted a qualitative descriptive design, appropriate for elucidating the complexities of participants’ experiential narratives and for delving into the myriad factors associated with specific phenomena, and has been widely used in the fields of health care and nursing [28]. The study employed one-on-one semi-structured in-depth interviews and adhered to the Consolidated Criteria for Reporting Qualitative Research (COREQ) checklist [29].

### Participants

This study employed a purposive sampling technique to identify and select participants who met the inclusion criteria. Purposive sampling allows for the inclusion of participants with diverse characteristics such as gender, education, and age, facilitating a comprehensive understanding and obtaining rich data [28]. The study primarily recruited Internet users among the elderly from the Shuangbei community, which is situated in the city center of Chongqing, the largest municipality in Southwest China. The internet penetration rate in Chongqing Municipality has exceeded 70%, which is comparable to the national average [30]. And as of the year 2020, the population aged 65 years and above accounted for 17.08% of the total population, which is higher than the average level of 13.5% in China [31]. The Shuangbei Community Health Service Center served as our educational collaborative community for this study. Prior to conducting interviews, the researchers contacted the person in charge at the community health service center and obtained their informed consent. The research plan and recruitment posters were provided to the individuals concerned to recruit sufficient participants. The inclusion criteria for the research participants were as follows: residents of the main urban area of Chongqing for at least 1 year, aged 60 years or older; engaged in various forms of Internet activities using internet-related devices such as smartphones, desktops/laptops, tablets, and internet-enabled televisions for a duration of at least 6 months; and willing to voluntarily participate in this study after giving informed consent. The exclusion criteria included: suffering from severe physical illnesses or being in an acute phase of illness; severe mental or psychological disorders; difficulty or lack of lucidity in language expression, or hearing impairment; and withdrawal from the study before completion.

### Data collection

Recruitment of participants and data collection were conducted from March to June in 2023. A semi-structured interview guide was designed and revised based on literature review, group discussions, and pre-interviews. The respondents were asked questions regarding the following areas: basic information about their Internet usage; their experiences and perceptions of using the internet; and their understanding of IA among older adults. (See more details in Table 1)

The interviews were conducted by a female doctoral student who had received training in qualitative research and refrained from providing any personal information to the participants. Consensus on the definition of IA used in this study was established with the participants prior to the interviews. Each face-to-face interview lasted approximately 30 to 60 min and took place in private and comfortable environments, such as the office of the community health service center or the participants’ homes, with only the interviewer and the interviewee present. With the informed consent of the participants, the interviews were audio-recorded synchronously with a mobile phone and voice recorder. The interviewer also performed field observations and documented non-verbal cues such as facial expressions, emotions, and body language exhibited by the interviewees to ensure the accuracy and reliability of the data. The number of participants in the study was determined based on the point of information saturation, which implies that interviews concluded when no new insights or perspectives regarding the interview topic and questions were obtained [32]. Data saturation was achieved in this study after interviewing the 36th participant. To confirm saturation and establish an adequate sample size, an additional two interviews were conducted, which corroborated that no new information was forthcoming. Consequently, the study proceeded with data analysis using a sample of 36 participants.

### Data analysis

Within 48 h after each interview, the audio recordings were manually transcribed into text. The transcriptions were then cross-referenced and supplemented with field notes and verification from the interviewees. Inductive content analysis was employed for data analysis in this study, a method especially suitable for exploratory research that does not aim to test theory [33]. The data analysis process consisted of four major stages [33]: (1) Sense of Whole, where four researchers (DW, XL, KC, YZ) read the interview data repeatedly to become thoroughly familiar with the overall content. (2) Open Coding, where two researchers (DW, XL) independently read the written material and recorded as many headings as necessary in the margins to describe all aspects of the content, generating categories freely at this stage. (3) Category Creation, where similar or related codes were compared and categorized, forming sub-categories. (4) Abstraction, which involved combining the sub-categories with similar contents into generic categories, such as individual, family, and peer factors, etc., which were subsequently combined into two main categories: risk and protective factors for IA in older adults. The resulting categories and corresponding quotes were independently translated into English by two bilingual researchers. Any discrepancies were discussed and resolved with the other bilingual researcher.

### Validity and reliability

This study employed various measures to enhance the credibility, transferability, dependability, and confirmability of the research. Firstly, all four researchers had received training in qualitative research methodologies during their university education and possessed experience in conducting interviews. They actively participated in the process of data analysis and interpretation. Secondly, in the interviews, the researchers thoroughly explained the research purpose to the participants and established rapport, aiming to obtain their full comprehension and trust. Thirdly, regular research team meetings were held to address any discrepancies arising from the data analysis process, until a consensus was reached on the main categories, generic categories, sub-categories, and corresponding quotes (representative statements from the interviewees). Lastly, to validate the authenticity of the findings, the researchers conducted discussions and cross-checks with the participants, comparing the interview data and results collected.

**Table 1:** Semi-instructured interview guideline

The interview questions
1. When and how were you first introduced to the internet? What are your preferred devices and online activities you engage in regularly and why?
2. What are the typical circumstances under which you use the internet? (For instance, context, time of day, and specific needs)
3. In your view, what role does the internet play in your life and how important is it?
4. What issues do you encounter when using the internet, or do you have any adverse online habits/annoyances/negative impacts? Please detail the process.
6. Do you perceive yourself to show any signs of IA? If so, please elaborate on the reasons, manifestations, and characteristics thereof. If not, please explain why.
7. How do you perceive the issue of IA among the elderly?

## Results

This study included interviews with a total of 36 elderly individuals, comprising 15 male participants (41.67%) and 21 female participants (58.33%). The average age among them was 68.64 years, ranging from 60 to 82 years. Among the participants, 26 had various chronic diseases, including coronary heart disease, diabetes, hypertension, cataracts, emphysema, etc. However, these conditions did not affect their cognitive abilities. The main online activities engaged by the participants included following online news, watching or creating short videos, online shopping, searching for health-related information, chatting and sharing on WeChat, online payments, mobile banking, reading online novels, playing online games (such as card and board games or Match & Eliminate game), and stock trading. The specific demographic information can be seen in Table 2.

The study extracted two main categories related to IA among elderly individuals: risk factors (including individual, family, peer, socio-environmental, and online platform factors) and protective factors (including individual and family factors). Please refer to Table 3 for a detailed analysis of the categories.

**Table 2:** Demographic information of participants

Variables	*N*
**Gender**	
Male	15
Female	21
**Age**	
60–69	22
70–79	12
80–89	2
**Education**	
Primary school and below	7
Secondary school	13
Senior high school	8
Technical Secondary School	3
University	5
**Pre-retirement occupation**	
University professor	1
Senior engineer	1
Staff/Civil servant	10
Worker	14
Peasant	6
Merchant/Unemployed	4
**Living Situation**	
Living with spouse	14
Living with children/grandchildren	16
Living alone	6
**Monthly income (CNY)**	
1000–3000	14
3001–5000	18
>5000	4
**Self-assessment of Internet usage duration per day (h)**	
2–3	14
4–5	9
≥ 6	13

**Table 3:** Factors for IA in older adults

Main categories / generic categories	Sub- categories
**1. Risk factors for IA in older adults**	
1.1 Individual factors	1.1.1 Internet as the major avenue for pursuing personal hobbies and interests.
	1.1.2 Excessive reliance on the pleasure and enjoyment derived from Internet use.
	1.1.3 Utilization of the Internet as the main pastime in an otherwise mundane life.
	1.1.4 Loneliness and psychological emptiness leading to overuse of the Internet.
	1.1.5 Habitual prolonged Internet use during instances of poor sleep quality.
1.2 Family factors	1.2.1 Household WIFI increasing the risk of prolonged Internet use indoors.
	1.2.2 Spousal Internet activities used as justification for one’s own Internet usage.
	1.2.3 Negative family dynamics leading to a predominantly Internet-centered lifestyle.
1.3 Peer factors	1.3.1 Peer recommendation and guidance as catalysts for intensified Internet engagement.
	1.3.2 Approval from peers regarding proficiency in Internet usage.
1.4 Socio-environmental factors	1.4.1 The widespread daily Internet use spurs offline intolerance.
	1.4.2 Internet’s facilitation of social interaction raises dependency risks.
1.5 Internet platform factors	1.5.1 The plenitude of online content triggers endless viewing/browsing behaviors.
	1.5.2 Incentive mechanisms of online platforms.
	1.5.3 Internet activities associated with addiction risks.
**2. Protective factors for IA in older adults**	
2.1 Individual factors	2.1.1 A higher level of perceived risk regarding internet health hazards.
	2.1.2 A stronger sense of self-control.
2.2 Family factors	2.2.1 More family commitment.
	2.2.2 Supervision and influence in multigenerational households.

### Main category 1 - risk factors for IA in older adults

The respondents shared their experiences and feelings about their IA, as well as their understanding and perspectives on the causes of IA. Based on this, we conducted a deductive analysis and identified potential factors contributing to IA across five dimensions: individual, family, peer, social environment, and online platform.

#### Individual factors

##### *Internet as the major avenue for pursuing personal hobbies and interests*

The Internet provides older individuals with better avenues and platforms to expand and fulfill their personal interests and hobbies. They engage in maintaining and developing their interests and hobbies through various online platforms such as social media and Douyin (a popular Chinese video-sharing platform, also known as Tik Tok). It is considered that this may also increase the risk of long-term addiction to the internet.

*N17: “I enjoy staying active in the WeChat group of our dance troupe on my smartphone, where we share information and updates. I also spend time watching dance videos on Douyin, as dance performance is my passion. It’s something I can’t seem to get enough of and engage in throughout the day.”*

##### *Excessive reliance on the pleasure and enjoyment derived from internet use*

Elderly individuals express a deep love for the online world. They thoroughly enjoy the pleasure that comes with being online and may even consider it as a manifestation of the meaning of life. This excessive enjoyment of being online can easily lead to excessive Internet use.

*N06: “I enjoy being online as it provides me with a sense of enjoyment. I don’t care about any drawbacks; I’m gonna keep enjoying being online! It’s what gives my life meaning, you know!”*

##### *Utilization of the internet as the main pastime in an otherwise mundane life*

The majority of respondents believe that, unlike the desire of young people for Internet use, IA among older adults tends to be more of a pastime in response to a mundane life.

*N20: “Unlike addiction among young people, I believe that addiction among older adults is a result of boredom. The use of smartphones to pass the time serves as a form of escapism, providing a sense of liberation.”*

##### *Loneliness and psychological emptiness leading to overuse of the internet*

Some elderly individuals, particularly those who live alone or have less involvement in family affairs, lack various avenues for communication and social interaction. As a result, they have more free time to utilize the Internet as a means to alleviate feelings of loneliness and spiritual emptiness.

*N16: “Due to loneliness and a lack of alternative means of communication, some elderly individuals may feel reluctant to engage in social interaction out of fear of being misunderstood. As a result, they turn to using smartphones as a means to pass the time and alleviate their feelings of solitude.” (A solitary elderly individual responded.)*

##### *Habitual prolonged internet use during instances of poor sleep quality*

Due to insomnia or early awakening, some elderly individuals tend to spend longer periods of time on the Internet during the night or early morning.

*N08: “I usually spend the most time on my smartphone in the wee hours, from around 2 AM to 5 AM. That’s because I often struggle with sleep issues and tend to wake up during that time. Playing on my smartphone has become a common activity for me during those hours.”*

#### Family factors

##### *Household WiFi increasing the risk of prolonged internet use indoors*

Most of the respondents expressed a preference for using the Internet in the comfort of their homes due to the availability of free wireless internet. Consequently, when outside, they opt for alternative forms of entertainment such as browsing or chatting, considering the limitations of mobile data usage. Some individuals even develop a habit of staying at home or instinctively going online whenever they are home, driven by the desire to access the internet.

*N03: “On a daily basis, I find myself engrossed in using my smartphone at home where WiFi is readily available and free of charge. However, when I venture outdoors, I am cautious about utilizing mobile data as it tends to deplete rapidly. Consequently, instead of indulging in smartphone activities, I tend to prioritize engaging in conversations with others outside of my home.”*

##### *Spousal Internet activities used as justification for one’s own Internet usage*

Many of the respondents, when describing their Internet usage patterns, also subconsciously mentioned their spouses, asserting that they often independently engage with their smartphones at home. Their tone and demeanor revealed a sense of psychological comfort derived from the fact that their partners also use the internet, which appears to justify their own online activities.

*N11: “At home, my spouse and I each indulge in our own online games. He enjoys playing ‘Chaos Chambers’ (a specific online game), while I prefer ‘Match and Eliminate’ (another online game).”*

*N12: “At home, it’s just my spouse and me. Like everyone else, he is constantly attached to his smartphone. He’s even juggling two smartphones simultaneously!”*

##### *Negative family dynamics leading to a predominantly Internet-centered lifestyle*

Elderly individuals exhibiting clear signs of IA often express that their children rarely visit them. Furthermore, they maintain a long-standing marital pattern of sleeping in separate rooms and avoiding interference in each other’s lives. Communication and interaction are minimal, making Internet usage a significant source of companionship in their lives.

*N06: “The children visit home only after long intervals, and my spouse and I have very little communication. We each indulge in our own smartphones, even after dinner. He sleeps in one room, and I sleep in another. It has been this way for more than a decade or two.”*

#### Peer factors

##### *Peer recommendation and guidance as catalysts for intensified internet engagement*

Respondents indicated that they were introduced to and learned some online activities by their peers and gradually became addicted to them in the process of using them.

*N01: “A good friend of mine, whom I have been friends with for many years, added me to a WeChat group where they sell products through live streaming. As a result, I find myself completely absorbed in watching these live streams every day.”*

*N02: “It was not my family who taught me, but my peers who showed me how to use smartphones and shop online. I usually spend my time on platforms like WeChat and Douyin, and the more I watch, the more interesting it becomes. However, this extensive screen time has resulted in my vision deteriorating and developing myopia.”*

##### *Approval from peers regarding proficiency in Internet usage*

Some respondents expressed that even though they spend a significant amount of time and effort online, engaging in activities they enjoy and find valuable, receiving recognition from friends boosts their confidence in continuing to use the Internet and improving their online skills.

*N27: “I believe I might be developing an addiction to the internet, primarily due to my involvement in tasks such as photo editing, video editing, and posting on TikTok. When I share these online activities with my friends, they seem to appreciate them. This makes me feel somewhat different from others and suggests that I might have room for improvement, even if it may simultaneously have some negative impacts on me.”*

#### Socio-environmental factors

##### *The widespread daily Internet use spurs offline intolerance*

Some respondents expressed that smartphones and the Internet have become essential tools in modern society. They have become so reliant on their smartphones that they cannot imagine being without them, and the thought of losing their smartphone and the associated access to the Internet is difficult to accept.

*N30: “Smartphones have taken the top priority in our lives. Without a smartphone, it feels like entering a “no man’s land”—a lifestyle that no one would rather prefer to be! We have become so accustomed to having smartphones that if they were suddenly taken away from us, it would be* worse than losing our lives, *and uncomfortable to go about our daily lives.”*

##### *Internet’s facilitation of social interaction raises dependency risks*

The internet breaks through spatial barriers and helps older people establish broader relationship networks and expand their social circles, holding a significant position in their lives. This may result in the elderly becoming overly reliant on online socialization, thereby neglecting face-to-face interactions offline.

*N18: Smartphones are quite important for me. I can’t seem to get enough of it! Through platforms like Douyin, I have been able to reconnect with classmates whom I haven’t seen in decades. I have also made new friends. Those who share the interest of using Douyin have become close companions, almost like sisters. Without smartphones, we would have gone our separate ways and lost contact with each other after all these years.*

#### Internet platform factors

##### *The Plenitude of Online Content triggers endless Viewing/Browsing behaviors*

The rich and diverse content available on the internet can cater to the various needs of older people, to the extent that they may find it difficult to disengage from spending extended periods of time online.

*N17: “I feel that online content is incredibly abundant! That’s why I carry a smartphone with me every day. I can’t stay disconnected even if it’s just for an hour.”*

##### *Incentive mechanisms of online platforms*

Some online software or activities use incentives such as economic rewards, preference identification, and personalized recommendations to encourage older adults to engage in relevant online activities, which may lead to addiction.

*N27: “My friends say that they see a few videos I post on Douyin every day because the app sends me timely reminders to upload content. Perhaps because I post frequently, I receive two reminders every day.”*

##### *Internet activities associated with addiction risks*

During this interview, it was found that activities that can potentially lead older adults to become addicted to the internet primarily include participating in online live streaming WeChat groups, being enthusiastic about inexpensive online shopping, and having a preference for online novels. Additionally, some respondents raised concerns about the risks of addiction to online gaming and gambling. Below are a few representative examples:

*N03: “I have a daily habit of buying discounted and reduced-price items on Douyin and Pinduoduo. For my strong addiction to shopping, my spouse scolds me, saying that I buy too much for the house!”*

*N27: “I used to be addicted to the internet mainly because of reading novels. Sometimes I would feel that videos strain my eyes, but I would wipe away my tears (from eyestrain) and continue watching. I would stay up until two or three in the morning, and even when I lay down in bed, my mind would still be consumed by thoughts of novels. The next morning, I would immediately pick up where I left off. As a result, my spouse and I would bicker over it at home.”*

### Main category 2 - protective factors for IA in older adults

The interviewee discussed their reasons for not getting addicted to the internet and how they made a determined effort to improve addictive behavior after realizing its negative impact. Based on this, we can deduce and analyze the potential protective factors against IA among older adults, which primarily encompass two levels: personal and familial factors.

#### Individual factors

##### *A higher level of perceived risk regarding internet health hazards*

The majority of the respondents expressed that due to their prioritization of personal health, they consciously avoided excessive internet usage. Moreover, upon experiencing health risks associated with internet usage, they exhibited determination to engage in self-improvement. Their commitment to maintaining good health triumphed over their desire for internet use.

*N12: “Since I developed cervical spondylosis and experienced the pain, I have been able to rein in internet usage.”*

*N24: “There is no need to indulge in the internet every day. Life is rich and colorful, beyond just smartphones. Health is undoubtedly important, as it belongs solely to oneself.”*

##### *A stronger sense of self-control*

Some older adults believed that their ability to avoid addiction stemmed from possessing a stronger sense of self-discipline. Moreover, they were able to quickly implement self-control measures upon experiencing negative consequences of IA.

*N24: “I am not addicted since I have strong self-discipline. Most people cannot stick to the routine of going to bed at 10 PM and waking up at 7 AM, but I have consistently adhered to this schedule for several years.”*

*N25: “I have a strong determination. For instance, if I stayed up until midnight using my smartphone yesterday, I make it a point to limit my usage to no later than 10 PM or 10:30 PM today. I firmly decide to turn off my phone and prioritize rest in order to avoid any disruption to my sleep.”*

#### Family factors

##### *More family commitment*

Some respondents indicated that their tight schedules involving numerous family responsibilities provided them with limited opportunities and circumstances for becoming addicted to the internet. Moreover, they possessed a strong sense of familial duty, often exercising self-restraint in order to prioritize completing their family obligations over excessive internet usage.

*N22: “Many factors prevent me from getting addicted because I believe in being responsible for my family. I consider addiction to be a dull and immature behavior.”*

##### *Supervision and influence in multigenerational households*

Family members’ reminders can assist elderly individuals in recognizing their harmful internet usage habits and behavior. This is especially true for older people living in multigenerational households who are concerned about how their internet activities may impact their grandchildren’s academic performance or serve as negative behavioral examples. As a result, they impose constraints on their own internet usage.

*N17: “My family members often remind me not to use my mobile phone excessively, especially in the evenings. Moreover, my grandson keeps an eye on my activities as well.”*

*N22: “At our current stage in life, which primarily revolves around taking care of our grandchildren, I realize that excessive phone use is not beneficial for them. Therefore, I now exercise control over my smartphone usage and refrain from excessive indulgence.”*

## Discussion

For all the substantial research on IA among young people, qualitative research in this area is limited. Furthermore, to our knowledge, there is currently no qualitative research specifically focused on IA among the elderly population. The primary objective of this research was to comprehensively understand the phenomenon and process of IA among the elderly by examining their perceptions and understanding of this burgeoning issue. This article aims to elucidate the factors of IA in the elderly. The findings of this study comprehensively elucidate the potential risk factors and protective factors associated with IA among the elderly, at various levels including individual, familial, peer, social, and online platform perspectives.

### Risk factors for IA in older adults

#### Individual factors

At the individual level, this study reveals that most older individuals with addictive tendencies enjoy using the internet because it allows them to explore and engage with content that aligns with their interests and hobbies. This finding is similar to a survey on IA among college students, where they identified the main purpose of internet usage as “fulfilling hobbies or specific needs” [34]. Internet services provide opportunities for older individuals to pursue an active and independent lifestyle, especially with the accessibility and affordability of user-friendly electronic applications and devices such as smartphones and tablets, which further broaden their engagement in online activities [35].

Additionally, some older individuals expressed a strong fondness for the internet and thoroughly enjoyed the pleasure it brings. This phenomenon may embody the psychological construct of flow experience [36], implying that profound enjoyment could potentially evolve into compulsive and/or addictive behaviors [37, 38]. However, compared to the fluctuating emotional experiences of enjoyment and pleasure, most participants mentioned that their motivation and experience of internet usage were more inclined towards leisurely pastimes in their daily mundane lives. This is consistent with the findings of other studies on younger demographics, including adolescents and college students [11, 39, 40]. With more leisure time, older individuals have access to vast resources on social media and other online platforms to seek entertainment and pass the time [41], helping them cope with or alleviate feelings of boredom. Furthermore, this study also demonstrates that loneliness is a potential cause of IA among older individuals, which aligns with other findings [17, 42, 43]. Due to reduced physical functioning and weakened social networks, older individuals are more susceptible to experiencing negative emotions such as loneliness [17], making them inclined to seek social interactions or entertainment through the internet to alleviate their loneliness.

Apart from emotional factors, the majority of the participants reported using the internet during sleep disturbances (insomnia or early awakening), leading to prolonged nighttime internet usage. This aligns with the limited existing research showing that sleep problems are predictors of IA [44, 45]. Additionally, a large-scale survey among older individuals confirmed a link between pre-sleep electronic device use and poor sleep quality [15]. Considering that older individuals are more likely to experience sleep disturbances or related disorders, many of them may choose to use the internet to fill the time when they struggle to fall asleep, but this, in turn, can exacerbate their sleep problems.

#### Family factors

This finding suggested that the IA behavior of older adults is influenced by their spouse’s internet usage. Similarly, recent research has found that parental IA is a significant predictor of adolescent IA [46, 47]. Similar to the role parents play in the lives of adolescents, spouses also play a crucial role in the lives of older adults. Therefore, the spouse’s internet usage issues can explain the relevant problems among older adults. This study also found that problematic family relationships could be a risk factor for IA among older adults, which is consistent with most other studies [47–50]. Additionally, this study revealed a new finding that goes beyond traditional research results, suggesting that having a WiFi-covered home environment may increase the risk of IA among older adults. Due to their tendency to be frugal, older adults perceive internet usage at home as more cost-effective and convenient, while being concerned about data usage costs outside. Within the permissible boundaries of both physiological and psychological health conditions, prompting older adults to eschew extended periods of domestic confinement and to engage suitably in extramural social events, as well as other indoor activities, may serve as an efficacious strategy for the prevention of IA within this demographic group.

#### Peer factors

Previous studies have primarily explored the relationship between negative peer relationships, such as deviant peer associations [51], peer bullying [52], and peer pressure [53], and IA. In contrast, this study proposes that the interest sparked in older adults through peer recommendations and guided learning of the internet may be a potential contributing factor to IA, highlighting the role of “positive peer relationships” in the process of internet use. We attribute this positive peer relationship effect among older adults to the explanation of the “collective clustering phenomenon,” where individuals with IA are more likely to communicate with like-minded individuals and may influence their friends in daily life to become internet-dependent [54]. Additionally, as “digital immigrants” who did not grow up in the internet era, older adults generally face digital skill deficiencies. Older adults who are relatively proficient in internet usage may be more susceptible to peer praise, leading to a sense of “digital superiority” and potentially disregarding negative consequences in their continued internet use, which may ultimately contribute to IA.

#### Socio-environmental factors

Participants expressed the significant role that smartphones, as the primary tool for internet use, play in their social lives. On one hand, smartphones have become a necessity that provides convenience in daily life to the extent that participants feel they cannot be without internet access. On the other hand, smartphones offer older adults easier and broader access to online socialization. This phenomenon is closely tied to the widespread adoption of smartphones globally, as they surpass other information and communication technology devices (including desktop computers, laptops, and tablets, etc.) in terms of portability, immediacy, and convenience [55, 56]. Consequently, smartphones have become the primary medium for accessing the internet and an integral component of contemporary life [55]. The increasing use of smartphones among the elderly is emerging as a common trend [57]. Diminished social roles and the deterioration of offline relationships may lead to greater reliance on smartphones among the older population, potentially resulting in addiction to these devices [55]. This bears many similarities to IA and promises to become a social issue of concern [55]. Furthermore, it is believed that this trend to some extent reflects the prevalent modern phenomenon known as “nomophobia,” which refers to the concern or fear of being unable to use or connect with others through smartphones [58]. As evidenced by participants using statements like “not having a smartphone is worse than losing one’s life” to describe the importance of smartphones to them. Of course, our research mainly reflects the attitude tendencies of older adults towards nomophobia. However, as older adults increasingly adopt and become proficient in using smartphones, their emotional gain from smartphones may increase, thereby increasing the risk of nomophobia [23]. Therefore, future research should specifically examine the characteristics related to nomophobia among older adults to obtain a more accurate analysis of the causes of IA in this cohort.

#### Internet platform factors

The majority of participants indicated that the abundant content available on the internet meets their diverse needs to such an extent that they find it difficult to stop using the internet even after prolonged periods, which is a major cause of IA. Moreover, online platforms incentivize older adults to continuously engage in related online activities, thereby leading to addiction. These findings are consistent with the findings of other studies explaining IA from a technical level [38, 59]. Online platforms, particularly short video platforms, with their user-friendly interfaces, captivating content, the opportunities for interaction with content creators and more precise and personalized content recommendations are likely to fully immerse elderly users and contribute to their endless consumption of online content [38, 59]. Additionally, future research should focus on the various subtypes of IA among the elderly, including health-related live streaming WeChat groups, online shopping, reading online novels, internet gaming and gambling.

### Protective factors for IA in older adults

#### Individual factors

The study obtained surprising and unique findings, which revealed that a higher level of perceived health risks associated with internet use may be more conducive to prevent or mitigate IA among elderly individuals. To the best of our knowledge, previous research has not explored the association between IA and the perception of health risks. However, a substantial body of evidence suggests that lower perception of smoking-related health risks is associated with higher initiation rates, longer duration of smoking, and fewer attempts to quit smoking [60–63]. Conversely, higher health risk cognition promotes the initiation or maintenance of positive health behaviors or the avoidance of negative health behaviors [64]. Kim et al. further confirmed that younger individuals are more prone to underestimating health risks [65]. Reasonable risk perception may increase with maturity due to increased exposure to health issues [66]. Based on these perspectives, we can hypothesize that elderly individuals, who have experienced more health problems, are more concerned about their physical well-being and are more likely to perceive the health risks associated with internet use or the negative health consequences of IA. However, this assumption is contingent upon elderly individuals understanding IA and being accurately aware of its negative consequences. Therefore, actively promoting health education related to IA can help elderly individuals enhance their awareness of the health risks associated with the internet, thereby preventing IA or prompting those inclined towards or experiencing IA to take timely measures.

However, merely being aware of the dangers of IA is not sufficient to avoid problematic internet use, individuals must also possess self-control. This finding is consistent with the majority of other studies [23, 42, 59, 67, 68]. Users with higher self-control are capable of avoiding or regulating the extent of their engagement in internet addiction-related behaviors. Elderly individuals generally exhibit higher levels of self-control [23], making them more likely to resist the urge to excessively use the internet or less likely to experience impulses or cravings related to the internet [42].

#### Family factors

Our research indicates that elderly individuals who have more family commitment are less prone to develop IA or are better able to control their addictive behaviors. This finding has not been previously reported in existing studies. However, this phenomenon can be understood and speculated from the perspective of “time management”. We believe that elderly individuals with higher levels of involvement in family affairs have less leisure time available, as they spend their days engaged in fulfilling activities without the need to rely on internet usage for time-passing purposes. Conversely, increased availability of leisure time and its inadequate management may contribute to excessive internet use [69, 70]. For elderly individuals, the increase in available time for internet usage, combined with their heightened sense of family responsibility, fosters a situation in which family members may actively engage in the development of suitable plans for familial matters. Such collaboration can be tailored to correspond with the preferences of the elderly, thus aiding in the enrichment of their quality of life and augmenting their perceived sense of worth.

Another protective factor is the supervision and influence of family members on the internet usage of elderly individuals. Consistent with previous research, certain studies have demonstrated the positive protective effect of parental involvement in family supervision against IA among children and adolescents [42]. However, this study emphasizes the significant role of multigenerational cohabitation in the family environment in preventing excessive internet use among elderly individuals. Specifically, elderly individuals, due to concerns about their online behaviors negatively influencing and demonstrating poor online conduct to their grandchildren, proactively restrict their own internet usage. Additionally, the supervision from grandchildren tends to be more effective in encouraging them to prevent or address their own problematic internet use, compared to the advice from their immediate children. This suggests that providing intergenerational support in a multigenerational cohabitation family environment may serve as an important strategy for preventing and intervening in IA among elderly individuals.

### Strengths and limitations

The potential misuse of the internet and the issue of IA among elderly individuals is becoming a new challenge for global public health. To the best of our knowledge, this is the first qualitative study specifically focusing on IA among the elderly population. This approach allows for a more authentic and comprehensive understanding of the existing issues related to internet use among the elderly and provides insights into the phenomenon of IA in this cohort. Moreover, it is aimed to raise awareness among elderly individuals about this emerging issue and promote healthy internet use through this study. Furthermore, the factors of IA among the elderly in detail from both risk and protective perspectives have been examined. Previous research has mainly focused on triggers and negative consequences.

However, this study has certain limitations. Firstly, the study participants were elderly individuals from Chongqing, a municipality in southwestern China. Due to differences in socio-cultural and economic development, some findings may differ from other countries. Secondly, our study does not draw definitive conclusions regarding the identification of IA in the elderly population. Therefore, following the development of a scientifically validated assessment tool for elderly IA, further investigative research should be conducted on individuals who meet the IA assessment criteria. Furthermore, given that the prevalence of IA among participants included in this study may be relatively low, future research should aim to enhance and refine our understanding by ensuring a balanced representation of both IA and non-IA elderly individuals and facilitating comparative analysis of interview data. Thirdly, the scope of the survey was somewhat limited. Future studies should aim to expand research areas and emphasize enhancing the transferability of findings to populations with experiences not sufficiently captured in the present study. Despite these limitations, we believe that this study is valuable as it enhances our understanding of the emerging phenomenon of IA among the elderly.

## Conclusions

This study employed a qualitative descriptive research approach to thoroughly investigate the factors associated with IA among elderly individuals. The behavior of IA in the elderly is influenced by multiple complex factors, including individuals, families, peers, social environments, and online platforms. One prominent innovative finding of this study is that having a higher level of perceived risk regarding internet health may be an important protective factor in effectively preventing and mitigating IA among the elderly. Additionally, strong self-control, more family commitment, and supervision and influence from multigenerational cohabitation households also play a preventive and buffering role in elderly individuals’ IA behavior. Based on these findings, it is well-justified that the “individual-family-peer-community” synergy strategy holds promise as an important direction for intervention in IA among elderly individuals. By implementing comprehensive prevention measures from multiple dimensions, we can effectively prevent the occurrence of IA among the elderly and promote healthy internet use.

## Data Availability

The data that support the findings of this study are available from the corresponding author upon reasonable request.

## Abbreviations

IA: Internet addiction
ADHD: Attention-deficit/hyperactivity disorder
